# Developing a psychological care competences framework for nurses in China: a mixed methods study

**DOI:** 10.1186/s12912-024-01778-3

**Published:** 2024-02-19

**Authors:** Qinghong Fang, Xingwen Li, Yuanyuan Luo, Zhihui Yang, Lin Xiao, Wenxuan Tan, Suting Liu, Jiahui Luo, Lili Zhang

**Affiliations:** 1 School of Nursing, Southern Medical University, Guangzhou, Guangdong Province China; 2 Department of Nursing, Taikang Tongji (Wuhan) Hospital, Wuhan, Hubei Province China

**Keywords:** Psychological care, Competence framework, Mixed-method approach, Delphi technique

## Abstract

**Background:**

With social transformation, rapid economic development and deepening awareness of psychological health in China, people’s demand for psychological health services is becoming increasingly urgent. A key challenge for Chinese medical organizations is to train enough qualified psychological care nurses. A greater understanding of psychological care competences (PCC) can help in clinical nurse selection, training, and assessment.

**Objective:**

To develop a PCC framework for Chinese nurses and obtain a consensus on the framework among experts.

**Methods:**

A descriptive mixed methods study was designed consisting of a literature review and semi-structured interviews followed by three Delphi rounds. The experts (*n* = 16) involved were nurses, nursing managers and educators from nine Chinese provinces with a specific interest in psychological care. Descriptive statistics assisted in data analysis.

**Results:**

Using the Iceberg Model as a theoretical foundation, five main dimensions and associated subdomains were integrated from 39 chosen articles. The semi-structured interviews with 24 nursing managers and nurses confirmed all of the themes from the literature review while generating new themes, both of which were incorporated into the initial PCC framework. After three Delphi rounds, the experts reached consensus on the PCC framework, including five domains (knowledge, skills, professional ethics, personal traits, internal motivations) and 22 subdomains with connotations. The response rate (RR) values for the three rounds of consultation were 80.00%, 87.50% and 92.86%, the composite reliability (Cr) values were 0.89–0.90, and the Kendall coordination coefficients were 0.155-0.200 (*P* < 0.05).

**Conclusions:**

On the basis of the Iceberg Model, literature review and qualitative research methods along with Delphi technique were used to develop a scientific and systematic PCC framework. The research methods were feasible and the results were reliable, thereby providing a basis for adopting this framework into nursing education. A formal assessment tool should be developed to test the PCC of nurses in clinical practice.

**Supplementary Information:**

The online version contains supplementary material available at 10.1186/s12912-024-01778-3.

## Background

Psychological problems have become a significant healthcare challenge. Approximately 300 million people worldwide experience psychological problems [1]. In China, 173 million people have mental health problems, accounting for 17.5% of domestic adults [2]. Compared to nonpatients, general hospital inpatients were reported to have significantly higher levels of anxiety and depression [3]. Approximately half of all patients admitted to general hospitals have a primary diagnosis of psychosomatic illness, while half of the remaining 50% have secondary or mixed psychological problems [4]. More worryingly, patients with physical health multimorbidity may have an elevated risk of suicidal behavior [5, 6]. Statistics show that there were 3.26 suicides per 100,000 nonpsychiatric inpatients in 48 Chinese general hospitals between 2015 and 2017 [7]. To handle psychological problems, domestic and foreign calls have been made to take mental health promotion actions as one of the main tasks and intervene in health influencing factors comprehensively [8, 9].

Psychological care, as a key component of holistic care, favorably enhances patients’ health outcomes [10, 11]. As the largest cohort of professionals that cares for patients and spends the most time with patients and families, nurses play a significant role in psychological care [12]. When patients exhibited psychiatric and psychological symptoms, their specific psychological condition was documented in nursing records, providing clinicians with prompt reminders [13]. What’s more, nurse-lead, interdisciplinary medical staff could give patients with timely and effective psychological care [13], as well as help refer patients to psychiatrists if necessary to meet their psychological requirements. Zhang et al [14] also emphasized that some patients may suffer from a higher risk of self-injury or suicidal behavior if not timely nursing intervened. Beyond that, nurse-facilitated psychological care may strengthen the lines of defense and resistance against the stressors faced by the patients throughout sickness and promote their psychological recovery [15]. However, Chinese nonpsychiatric nurses now have inadequate psychological care competence compared to psychiatric nurses, resulting in high rates of underdiagnosis of patients’ psychological problems [16]. In addition, some Chinese nurses lack psychological care knowledge and skills, which makes it difficult for them to independently solve psychological problems [17]. For the above reasons, the Chinese psychological care competence of nonpsychiatric nurses needs to be improved.

The nursing competence framework is used as a reference for promoting clinical nursing development and orienting professional practice standards [18]. Psychological care competences (PCC) are multidimensional, including knowledge, skills, self-concept, motivation, and characteristics [19]. Many countries have established professional PCC frameworks. King et al. [19] identified the existing competence sets related to disaster mental health through a literature review. Carroll et al. [20] explored midwives’ competency in perinatal mental health by exploratory descriptive study. Waite et al. [21] reviewed the definition of structural competence in mental health care. Because competence frameworks are intended for practice in specific contexts, direct adaptation of PCC frameworks from other countries in China may be inappropriate due to cultural and healthcare system differences. Hence, it is necessary to develop a PCC framework to provide comprehensive guidance for Chinese nonpsychiatric nurses.

For the development of a competence set, researchers commonly use methods and theories. It is typical to seek expert perspectives to identify a consensus stance because expert opinions are thought to be more reliable than others [22]. The Delphi methodology is one way to build a competence framework, utilizing anonymous sequential questionnaires to achieve a consensus among ‘experts’ [23]. The exchange of opinions across several rounds promotes Delphi consensus [24]. This collective review by a representative group is appropriate for competence framework development [23]. Meanwhile, the Iceberg Model, proposed by the American psychologist Spencer [25], can be used to direct the construction of competency frameworks. Therefore, based on the Iceberg Model, a mixed-method approach was used in this study.

The aim of this study was to (1) develop a PCC framework for Chinese nurses and (2) obtain a consensus on the framework among experts.

## Methods

### Design

**Fig. 1:**
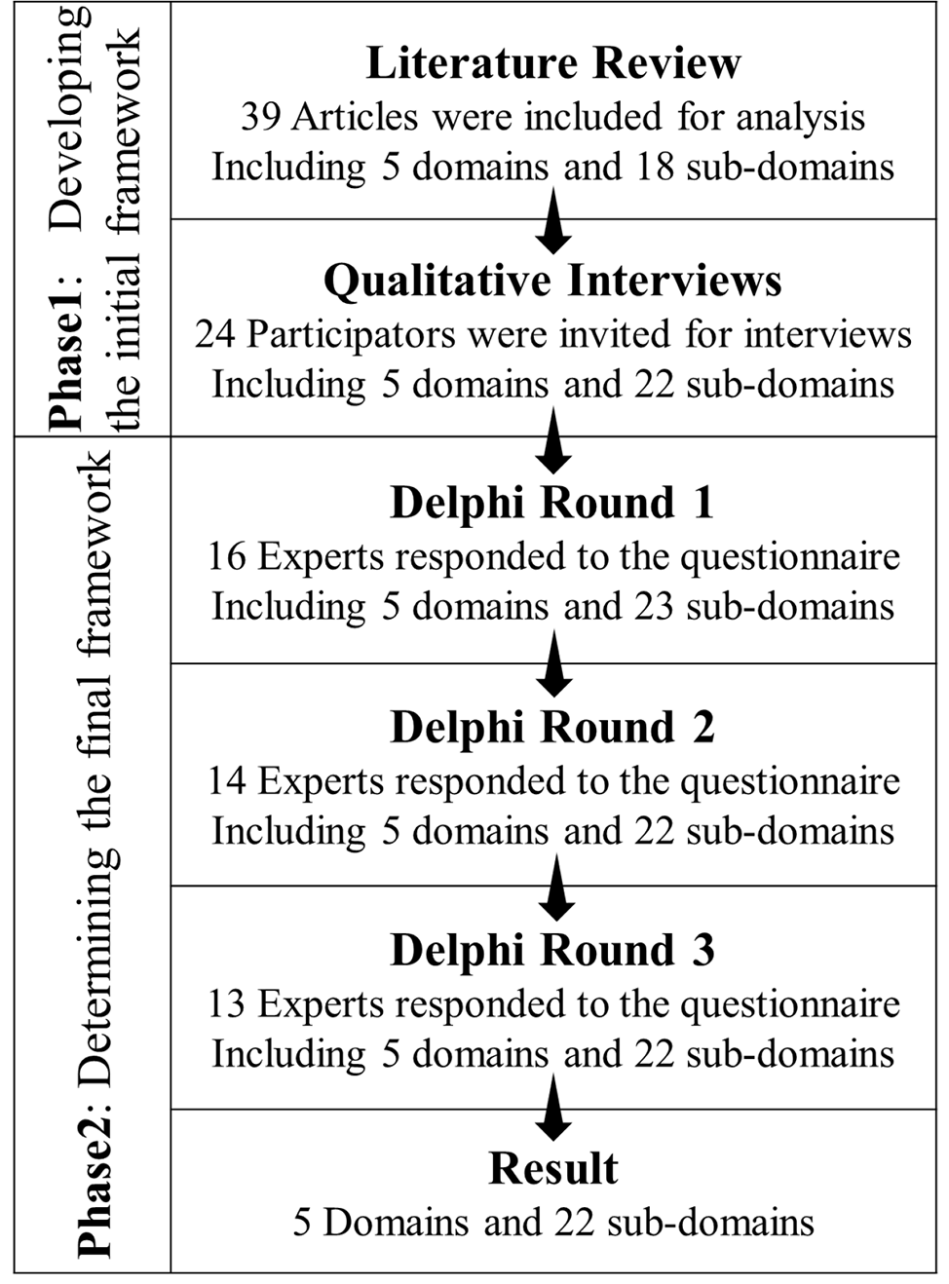
Summary of research design in developing competence framework for psychological care

This study was designed in two phases using a mixed-method approach that included literature review, qualitative study and Delphi technique, as shown in Fig. 1. The first phase developed the initial psychological care competences (PCC) framework, in which PCC characteristics were extracted through literature review and qualitative interviews. Five competence domains and 22 sub-domains were identified. In the second phase, the framework was revised through Delphi rounds until a consensus was reached. This report was checked against the Conducting and REporting of DElphi Studies (CREDES) [26].

### Research team

The team comprised eight members: one professor supervised the overall planning of the study, three associate professors were responsible for supervising the research process, and four nursing graduate students were in charge of researching the specific implementation procedure.

### Phase 1: development of the initial PCC framework

#### Literature review

The search strategy was developed collectively by two writers (QHF, XWL) who had received systematic review training, and the search terms were determined after discussion by the research team. The electronic databases PubMed, Embase, Cochrane Library, CINAHL, PsycTESTS, PsycArticles, PsycInfo, Psychology and Behavioral Sciences Collection, CNKI, Chinese Biomedical Literature Database, and Wanfang were searched from their earliest dates to October 2022. A mix of medical subject headings and free text terms of the following key concepts was used for the search strategy: nurs*, midwi*, APN, NP, psychological car*, psychological nurs*, depression management, stress management, mental health car*, mental health service*, competenc*, self-competence, abilit*, skill*, knowledge*, attitude*, behavio* (Appendix 1).

The team decided on the inclusion criteria as follow: (1) nonpsychiatric nurses were the primary focus of the articles, (2) the research content provided a clear definition of nurses’ PCC, (3) the outcome indicators were nurses’ competences to perform psychological care or the development of nurses’ PCC assessment tools, (4) the research types included quantitative, qualitative, mixed-method and descriptive papers and various types of reviews, (5) articles were written in Chinese and English. Literature exclusion criteria were (1) repeated papers, (2) articles for which full text could not be obtained.

A total of 27,266 records were retrieved and imported into EndNote X8. After removing duplicates, 19,245 articles remained. The first author (QHF) screened the titles and abstracts and excluded irrelevant papers according to the inclusion and exclusion criteria, and 327 articles were included for full-text screening. Next, two authors (QHF, XWL) reviewed the full texts of the potentially relevant articles. Any disagreements about inclusion were resolved through discussion, and if needed, the third author (YYL) was consulted to reach a consensus. Finally, 39 articles were included in this study.

The directed content analysis approach [27] was used to extract elements related to PCC from the literature. Based on the Iceberg Model, competency elements were classified into different themes, such as knowledge, skills and personal traits [28–29]. The team discussed and combined each individual element into the framework.

#### Qualitative interviews

From November 2022 to February 2023, semistructured interviews were conducted to supplement PCC. Purposive sampling was used for participant recruitment. The inclusion criteria were willingness to participate in the study, holding a bachelor’s degree or above in nursing, and having at least five years of clinically psychological care experience in nonpsychiatric wards. In this research, 24 participants from four comprehensive hospitals in southern China were recruited.

A structured guide was initially developed based on the STAR (S = situation, T = task, A = action, R = result) principle of the Critical Incident Technique (CIT) to effectively extract PCC and prevent interviewees from going off-topic during the conversation. Then, one nursing manager and one nurse who met the participant inclusion criteria were invited to pretest the guide. The interview guide was revised and finally determined through a discussion among the team, mainly covering the opinions and experiences of psychological care (Appendix 2).

After receiving informed consent from the participants, interviews were held and recorded. The interviews were then transcribed verbatim within 48 h. Two experienced researchers (QHF, XWL) repeatedly read the data and used the directed content analysis approach [27] to analyze the data through NVivo 11.0. First, the meaning of sentences significant to the topic were outlined. The initial codes were then extracted and grouped into categories and subcategories based on similarities and differences. Finally, the description of each category cluster was repeatedly compared to reflect the Iceberg Model and the framework subsequently was refined. Any discrepancies were resolved by discussion and consensus with the research team.

### Phase 2: delphi rounds to determine the final PCC framework

#### Panel of experts

Purposive sampling was used to recruit experts. The inclusion criteria for experts included: (1) work experience: engaged in psychology care, nursing practice, nursing management, or nursing education with psychology care research experience; (2) educational background: held at least a bachelor’s degree; (3) professional title: intermediate and above; (4) work years: had worked for 5 years or more; and (5) provided informed consent. The minimum sample size for the Delphi technique is 10–15 participants [30]. With the help of corresponding author (LLZ), the team obtained the e-mail addresses of 20 experts from ten Chinese provinces. Ten experts were from hospitals, and the others from schools. We sent invitation e-mails to them at the same time.

#### Data collection

The three-round surveys were conducted between March and May 2023. There were three parts in the survey questionnaire: (a) Introduction: This section briefly explained the study’s purpose, voluntary nature, and confidentiality procedure; (b) The initial PCC framework: This section included 5 domains and 22 subdomains proposed in phase 1. Participants were asked to rank the importance of each item on a 5-point Likert scale ranging from 1 (not at all important) to 5 (very important). They were also allowed to comment on each competence and propose new competences; (c) Demographic characteristics: This section included basic information about the experts, such as age, gender, educational background, work direction, professional title, seniority, institution, familiarity coefficient (Cs), and judgment coefficient (Ca). The Cs data were separated into five levels (0.20 = unfamiliar, 0.4 = less familiar, 0.6 = generally familiar, 0.8 = very familiar, 1.0 = extremely familiar), and the Ca data were divided into four categories: theoretical analysis, literature reference, practical experience, and subjective judgment.

The experts received questionnaires through e-mail, and they were expected to complete the questionnaires within one week. The team collected and analyzed the results of the expert questionnaire responses. Competence items reached consensus when the mean was > 3.50 (the importance of items), the coefficient of variation (CV) was < 0.25 and ≥ 75% of respondents rated an item as 4 or 5 points [31–32]. Competence items that did not reach consensus in the first round were modified, deleted or added to the statement and subsequently were fed back in the second round, allowing the experts to potentially revise their responses based on the opinions of other experts. After reviewing the results from the second round, the team later used the feedback to modify the questionnaire for the third round. The updated questionnaire was given to experts in the same manner as before. Experts were asked to complete the revised questionnaire in the same way as before. Following the Delphi guidelines, Delphi rounds need to be repeated until an agreement is reached [26]. A consensus was reached after the third Delphi round. To preserve the privacy of participants, the team ensured that during the surveys, participants did not meet or know the identities of other participants, and the questionnaires were given to each participant individually [33].

#### Data analysis

Excel 2019 and IBM SPSS version 25.0 were used to analyze the quantitative data. Descriptive analysis was conducted using frequency, proportion, mean and standard deviation. The recovery rate (RR) and the authority coefficient of experts were calculated to test the reliability and accuracy of expert consultation results. The calculation formula of the authority coefficient (Cr) is Cr=(Cs + Ca)/2 [34]. The consistency among experts was presented by the coefficient of variation (CV) and Kendall coefficient (W). The difference was statistically significant with *P* < 0.05 when the chi-square test was applied for significance analysis. In addition, Yaahp version 10.3 was used to build a model with two levels corresponding to the competence framework [35]. Based on the third-round value judgment of the experts, the importance weights of each item were calculated by using the analytic hierarchy process (APH) method. For consistency testing, the consistency index (C.I.) and average random consistency index (R.I.) are usually adopted, and the ratio of C.I. and R.I. is called C.R. When the consistency ratio of the judgment matrix is CR < 0.10, the judgment matrix is considered to have satisfactory consistency [36].

#### Quality control

The validity of the questionnaire responses in each round was double checked. After receiving the questionnaires, team members separated the expert personal information forms and the consultation forms into two different folders. The consultation forms did not include expert names, ensuring that expert opinions were anonymous during data analysis. Furthermore, to ensure external validation of the results, the final PCC framework was reviewed and approved by four external experts (two professors from medical universities and two specialist nurses from tertiary hospitals).

### Ethical considerations

This study adhered to the tenets of the Declaration of Helsinki. The university affiliated hospital’s ethics committee gave its approval for the study (ID: NFEC-2023-037). Informed consent was provided and obtained from all participants before the study commenced.

## Results

### Literature review and qualitative interviews

After literature screening, the 39 included articles were finally analyzed. At this stage, we formed the first draft of the PCC framework, including 5 domains and 18 sub-domains.

In the stage of qualitative interviews, 24 participants took part in the interviews with a mean age of 39.67 ± 6.80 years old and a mean working time of 18.20 ± 8.32 years from fields of psychological care, nursing education and nursing management. The demographic characteristics of these participants are shown in Table 1. The prior report offered more details about the interview results [37].

Based on the results of the literature review and semi-structured interviews, the team formed the initial PCC framework, including 5 domains and 22 subdomains: knowledge (knowledge of nursing psychology, knowledge of clinical nursing, knowledge of humanities and social sciences), skills (psychological care practical skills, clinical nursing practical skills, psychological crisis management skills, interpersonal skills, health education skills, thinking skills), professional ethics (self-development, responsibility, empathy, equal and respect, professional attitudes, legal awareness), personal traits (self-mental quality, self-reflection, character quality, self-image) and motivations (psychological care awareness, sense of professional identity, sense of collective honor).

**Table 1:** Qualitative interviews participants’ demographic characteristics

Characteristic	Value
Age (year), $\overline x \pm s$ (range)	39.67 ± 6.80 (29–56)
Work experience (year), $\overline x \pm s$ (range)	18.20 ± 8.32 (6–36)
Professional title, *n*	
Nurse	3
Nurse in charge	15
Associate senior nurse	2
Senior nurse	3
Nursing administrator	1
Education level, *n*	
Bachelor’s degree	20
Master’s degree	4
Gender, *n*	
Female	24
Male	0
Ward, *n*	
Oncology	6
Radiotherapy	4
Galactophore	3
Orthopedic	2
Endocrinology	2
General surgery	2
Neonatal intensive care unit	1
Hepatobiliary surgery	1
Traditional Chinese medicine	1
Gastroenterology	1
Administrative unit	1

### Positivity, demographic characteristics, authority and coordination of experts

The response rates of experts for the three Delphi rounds were 80.00% (16/20), 87.50% (14/16), and 92.86% (13/14). These experts came from seven hospitals and six universities in nine Chinese provinces (Guangdong, Sichuan, Beijing, Tianjin, Henan, Shanxi, Hunan, Shanghai, and Gansu). The demographic characteristics of these participants are shown in Table 2. The Cr scores for the three Delphi rounds were 0.89 (Ca = 0.94; Cs = 0.84), 0.89 (Ca = 0.94; Cs = 0.84) and 0.90 (Ca = 0.94; Cs = 0.85), respectively. Table 3 displays the degree of coordination among experts in each round.

**Table 2:** Delphi participants’ demographic characteristics

Characteristics	Round 1(*n* = 16) n (%)	Round 2(*n* = 14) n (%)	Round 3(*n* = 13) n (%)
Age (years)			
< 40	2 (12.50)	2 (14.29)	2 (15.38)
40–50	8 (50.00)	6 (42.86)	5 (38.46)
> 50	6 (37.50)	6 (42.86)	6 (46.15)
Gender			
Male	1 (6.25)	1 (7.14)	1 (7.69)
Female	15 (93.75)	13 (92.86)	12 (92.31)
Educational background			
Bachelor’s degree	5 (31.25)	3 (21.43)	3 (23.08)
Master’s degree	6 (37.50)	6 (42.86)	5 (38.46)
Doctorate	5 (31.25)	5 (35.71)	5 (38.46)
Work direction			
Nursing education	6 (37.50)	5 (35.71)	5 (38.46)
Nursing management	4 (25.00)	4 (28.57)	4 (30.77)
Psychology	6 (37.50)	5 (35.71)	4 (30.77)
Professional title			
Intermediate (Lecturer)	2 (12.50)	2 (14.29)	2 (15.38)
Associate senior (Associate professor/Associate senior nurse)	7 (43.75)	5 (35.71)	5 (38.46)
Senior (Professor/Senior nurse)	7 (43.75)	7 (50.00)	6 (46.16)
Seniority (years)			
5-<20	4 (25.00)	3 (21.43)	3 (23.08)
20–30	5 (31.25)	4 (28.57)	4 (30.77)
> 30	7 (43.75)	7 (50.00)	6 (46.15)
Institution			
Hospital	8 (50.00)	7 (50.00)	6 (46.15)
University	8 (50.00)	7 (50.00)	7 (53.85)

**Table 3:** Delphi participants’ coordination degree

Items	Number(***n***)	Mean^i^	SD^i^	Items ≥ 4 points(%)^i^	CV^i^	Kendall’s W	χ^2^	***P***
Round 1								
All items	27	3.88 ~ 5.00	0.00 ~ 1.36	62.50 ~ 100.00	0.00 ~ 0.35	0.184	76.418	< 0.001
First level	5	4.50 ~ 4.94	0.25 ~ 0.73	87.50 ~ 100.00	0.05 ~ 0.16	0.175	11.186	0.025
Second level	22	3.88 ~ 5.00	0.00 ~ 1.36	62.50 ~ 100.00	0.00 ~ 0.35	0.197	66.051	< 0.001
Round 2								
All items	28	4.50 ~ 5.00	0.00 ~ 0.65	92.86 ~ 100.00	0.00 ~ 0.14	0.155	58.660	< 0.001
First level	5	4.71 ~ 5.00	0.00 ~ 0.61	92.86 ~ 100.00	0.00 ~ 0.13	0.185	10.341	0.035
Second level	23	4.50 ~ 5.00	0.00 ~ 0.65	92.86 ~ 100.00	0.00 ~ 0.14	0.154	47.534	0.001
Round 3								
All items	27	4.62 ~ 5.00	0.00 ~ 0.51	92.31 ~ 100.00	0.00 ~ 0.14	0.200	67.589	< 0.001
First level	5	4.70 ~ 5.00	0.00 ~ 0.48	100.00	0.00 ~ 0.10	0.195	10.154	0.038
Second level	22	4.62 ~ 5.00	0.00 ~ 0.51	92.31 ~ 100.00	0.00 ~ 0.14	0.205	56.032	< 0.001

^i^: The minimum ~ maximum value of the importance of items

### Delphi round 1

Thirteen experts (81%) put suggestions in the first round. Items were modified or added based on experts’ opinions. In the first-level competencies, “motivations” was revised to “internal motivations”. In the second-level competencies, “thinking skills” was revised to “thinking cognitive skills”, “legal awareness” was revised to “legal and ethical awareness”, “self-mental quality” was revised to “mental health literacy”, “self-reflection” was revised to “self-reflective habit”, “sense of professional identity” was revised to “sense of professional acquisition”, and “sense of collective honor” was revised to “sense of collective belonging”.

Three experts suggested that the item “self-development” should be moved from “professional ethics” to “personal traits”. It was accepted with a reason stated by one of the experts, the realization of “self-development” indicates the evolution of an individual’s inner thinking and belongs to personal quality from the psychological point of view.

An expert suggested that “physical health literacy” should be added to the second level of “personal traits”. We accepted the suggestion that nurses’ own physical health is as important as their mental health.

### Delphi round 2

In the second round, the altered PCC framework after the first round was presented to the experts. Six experts (43%) made revision suggestions, with 6 of the 23 items requiring further revision. In the second-level competencies, “mental health literacy” was revised to “psychological quality”, and “physical health literacy” was revised to “physical quality” because one expert pointed out that “health literacy” involves knowledge and skills that are inappropriate for the domain of personal traits. The team unanimously accepted this suggestion. In addition, “self-image” was revised to “etiquette and image”, and “psychological care awareness” was revised to “psychological care concept”.

Some modifications were made to more effectively direct the development of the PCC framework. Two experts strongly suggested deleting “character quality” because character is not to be distinguished as good from bad, and this item was repeated in the domain of professional ethics. After a discussion, the team consistently agreed to delete this item. One expert suggested changing the “legal and ethical awareness” to the “legal and ethical practical skills” and reorienting this item to the domain of skills because “practical skills” could be more specific to guide training content.

### Delphi round 3

The opinions of the experts converged in the third round, and only one amendment was proposed. “Legal and ethical practical skills” was revised to “legal practical skills”. At the end of the third round, five domains, 22 subdomains, and connotations of the PCC framework obtained a consensus of 75% or more among the expert panel. The weights of each item were determined with a combination of the Delphi technique and the APH method, and all items reached CR < 0.10, as presented in Table 4.

**Table 4:** Importance scores, CV, weights and connotations for each item

Items	Mean ± SD	CV	Weights	Connotations
1 Knowledge	5.00 ± 0.00	0.00	0.205	Having a good knowledge of nursing, psychology and related humanities, which are required for clinical care.
1.1 Knowledge of nursing psychology	5.00 ± 0.00	0.00	0.316	Proficient in basic concepts, theories and knowledge of nursing psychology and psychiatric psychology.
1.2 Knowledge of clinical nursing	5.00 ± 0.00	0.00	0.342	Proficient in basic theories of clinical nursing and principles of nursing based on the theories of medical profession.
1.3 Knowledge of humanities and social sciences	4.62 ± 0.51	0.11	0.342	Familiarity with knowledge of humanities and social sciences related to health and care, and socio-cultural factors affecting patients’ psychological and physical health.
2 Skills	5.00 ± 0.00	0.00	0.205	Be able to perform psychosomatic nursing tasks such as emotional management, nursing practice, health education, interpersonal communication, and legal practice to meet the psychosomatic needs of patients and their families.
2.1 Psychological care practical skills	5.00 ± 0.00	0.00	0.138	Be able to comprehensively assess the psychological status of patients and their families with scales or other methods, to pose psychological care questions, to implement individualized psychological care through listening, reassurance or encouragement, and finally to evaluate the effects.
2.2 Clinical nursing practical skills	5.00 ± 0.00	0.00	0.140	Be able to grasp basic and specialized nursing techniques, to complete various nursing work steadily, quickly and accurately, thereby reducing patients’ suffering.
2.3 Psychological crisis management skills	4.92 ± 0.28	0.06	0.138	Be able to notice patients in psychological crisis, to identify situations that patients need referral, and to assist in providing psychological services like professional accompaniment and conversation.
2.4 Interpersonal skills	5.00 ± 0.00	0.00	0.144	Familiarity with social skills such as communication, coordination and cooperation with patients, their families or co-workers, as well as adaptability to situations like competition, praise or criticism at work.
2.5 Health education skills	4.69 ± 0.48	0.10	0.144	Proficiency in health-information dissemination skills to help patients and their families rise health awareness and mobilize patients and their families to participate in nursing activities.
2.6 Thinking cognitive skills	4.77 ± 0.44	0.09	0.147	In clinical practice, with own knowledge and experience, be able to comprehensively analyze and orderly reason patients’ existing or potential psychological problems, and ultimately make the appropriate judgment and choice.
2.7 Legal practical skills	4.69 ± 0.48	0.10	0.147	Able to deeply understand the importance of various regulations and practical procedures; to consciously fulfill nurses’ legal obligations; to protect nurses’ legitimate rights and interests as well as those of the hospital through the law.
3 Professional ethics	4.92 ± 0.28	0.06	0.202	The ethical qualities required for the implementation of psychological care in hospitals are manifested as responsibility, empathy, dedication and so on in the nurses’ psychological care services.
3.1 Responsibility	5.00 ± 0.00	0.00	0.250	Could assume responsibility for meeting the physical and psychological needs of patients and their families, conscientiously observe professional norms and fulfill professional obligations; proactively safeguard patients’ right of life, privacy and informed consent.
3.2 Empathy	5.00 ± 0.00	0.00	0.250	Could perceive, grasp and understand the thoughts of patients or their families.
3.3 Equal and respect	5.00 ± 0.00	0.00	0.250	Regardless of the differences in social status, living conditions, job, cultural background, and religious beliefs among the nursing clients, nurses treat them equally, and similarly, nurses respect their different individuality and personality.
3.4 Professional attitudes	5.00 ± 0.00	0.00	0.250	Could hold cognition, affection, and behaviour required by psychological care, including the degree of effort and proactivity, as evidenced by carefully caring for patients and/or their families, patiently explaining knowledge related to care, and genuinely interacting with patients and/or their families.
4 Personal traits	4.69 ± 0.48	0.10	0.192	The personal traits are manifested as having a stable mood, healthy physique and decent demeanor, which could positively influence patients and their families and then alleviate their negative feelings.
4.1 Psychological quality	5.00 ± 0.00	0.00	0.208	Internalize externally acquired stimuli into stable, fundamental and implicit mental qualities that have derivative and developmental functions.
4.2 Physical quality	4.92 ± 0.28	0.06	0.205	Stay energized to provide services to patients or their families through appropriate exercise, good sleep, and healthy eating.
4.3 Self-development	4.77 ± 0.44	0.09	0.198	Self-grow to improve nursing knowledge and skills in psychological care.
4.4 Self-reflective habits	4.77 ± 0.44	0.09	0.198	Develop the habit of self-reflection by reviewing and learning the experience of psychological care services.
4.5 Etiquette and image	4.62 ± 0.65	0.14	0.192	Dress neatly and behave elegantly, then use the beauty of nurse’s professional image to warm the patients and their families.
5 Internal motivations	4.77 ± 0.44	0.09	0.196	To promote the mental health of patients and their families, internal motivations are used to stimulate and maintain psychological nursing care behaviors.
5.1 Psychological care concept	5.00 ± 0.00	0.00	0.344	Clarify nurse’s role ideologically, fully recognize the importance of psychological care, and then implement psychological support, psychological nursing or mental health education for patients.
5.2 Sense of professional acquisition	4.85 ± 0.38	0.08	0.333	Feeling rewarded by nurse’s profession at work, seeing value in psychological care, and maintaining a positive emotional state.
5.3 Sense of collective belonging	4.69 ± 0.48	0.10	0.323	A feeling when being recognized and accepted collectively can motivate enthusiasm to nursing and thus to be proactive in providing psychological care.

## Discussion

Combined with the results of literature review and qualitative interviews, this study preliminarily formed a comprehensive and multi-perspective description of the PCC framework, providing strong evidence support materials for further Delphi studies. This was consistent with the point made by Guo et al [38]. After three rounds of Delphi, the PCC framework included five domains (knowledge, skills, professional ethics, personal traits and internal motivations) and twenty-two subdomains.

For the study to be scientific and reliable, it is crucial that the experts chosen for the Delphi method should be represented, motivated, and authoritative [39]. First, the experts involved in this study had solid professional knowledge and rich work experience. They came from nine Chinese provinces and had worked for five or more years in nursing or psychological fields. Second, a questionnaire RR > 70% was regarded as an effective consultation [40]. All RRs of the three rounds were effective. Meanwhile, the experts provided constructive comments on the framework, suggesting that the experts were enthusiastic and active in the Delphi rounds. Third, the Cr scores in the three Delphi rounds were > 0.80, and Kendall’s W for the three rounds was statistically significant (*P* < 0.05), indicating that the results are acceptable and reliable [34]. Finally, external experts verified the final PCC framework, enhancing the validity of the research results.

A competency framework is developed to support healthcare development and ensure consistent high standards of care nationally [41]. The PCC framework, developed according to the native culture, could direct nursing administrators or educators to design competence-based training programs [42]. More importantly, this framework may promote clinical psychological care practices and improve mental health services.

Among the first-level items, the average score and the weight value of “knowledge” are the highest. Knowledge, as part of the iceberg above, is the most basic competence in psychological care. Nurses need to master the theory of psychological care, the identification of adverse emotions, the maintenance of mental health [43], and clinical nursing expertise. Additionally, nurses need to know well about humanities and social sciences [21, 44]. Knowledge plays an important role in competence development [45]. As early as Ancient China, there was the idea that knowledge precedes practice [46]. Meanwhile, the Knowledge-Attitude-Belief-Practice (KABP) model in Western countries believes that knowledge is the basis for changing behavior [47]. In the field of psychological care, nurses’ behavior is related to the requisite knowledge, skills, and work experience [48]. It has been shown that knowledge is positively related to PCC and that nurses are more capable of providing services when they have a higher level of knowledge [49–50]. Therefore, to accommodate the future development of psychological care, nursing administrators need to conduct various trainings about PCC, invite psychological experts to give lectures and then strengthen nurses’ awareness of psychological care.

The weight of “skills” is comparable to that of “knowledge”, suggesting that skills give significant play to the PCC. Some items in skills, such as practical psychological care skills, practical clinical nursing skills, psychological crisis management skills and interpersonal skills, are consistent with previous findings in other countries [51], indicating that experts’ expectations of skills in Chinese PCC are in line with international standards. It is noteworthy that thinking cognitive skills and legal practice skills have the highest weights among the second domains, which may be related to the professional requirements for Chinese nurses. The Chinese standardized training program for new nurses encourages nurses to assess evidence-based evidence using critical thinking and scientific methods, thus enhancing their professional knowledge and ability [52]. Moreover, research indicates that enhancing practical legal skills of nurses could ensure patient safety and decrease care conflict [53]. Hence, to increase nurses’ psychological care skills, healthcare institutions could try to devise unique trains on various psychological care topics.

Each item on the dimension “professional ethics” has a weight of 0.250, suggesting that they are all equally essential. “Professionalism and ethical behavior are central to medical practice” [54]. Additionally, the realization of the Healthy China strategy calls for a large number of highly ethical medical professionals. For the item “equal and respect”, a previous study similarly suggested that nurses should respect all patients equally when communicating with them [55]. In addition, Chinese Medical Education believes that a positive professional attitude is the basic requirement for medical professionals [56]. Nurses’ competence to care for patients would be severely hampered if they treat patients poorly at work and lack empathy [57]. Enlighteningly, to direct nurses think about ethics dialectically and logically and to regulate their own roles and actions, hospitals need to carry out clinical psychological care practices from an ethical perspective. Of course, nurses themselves should also combine scientific skills with humanistic literacy.

The weight of “psychological quality” in the dimension of “personal traits” is 0.208, ranking first. Nurses are one of the most important forces in national health care, and their physical and mental health is a guarantee for patients’ life safety and mental health [58]. Nurses with mental health problems have a detrimental impact on their personal health and have a negative effect on the quality of nursing services and patient treatment [59–60]. In addition, both “self-development” and “self-reflective habits” have weights of 0.198. When developing the nursing competency framework, scholars from many countries believed that the nursing profession aimed to enhance care quality and provide more person-centered care through lifelong learning and reflective practic [32, 61, 62]. In this study, the experts advised that hospitals should create a humanized working atmosphere, pay attention to the physical and mental health of nurses, and be concerned about nurses’ personal career development aspirations.

“Psychological care concept” is ranked first in the dimension “internal motivations”, with a weight of 0.344. In the practical actions of socialist modernization, developing and adhering to correct values can help people perform duties [63]. Nurses who construct the concept of psychological care could keep themselves involved in high-quality psychological care. The item “sense of professional acquisition” is offered in the context of Chinese comprehensive reform, which aims to improve the medical care system and realize shared development [64]. Nurses’ sense of professional acquisition refers to the positive emotional state of being content with their career because they enjoy it and value it [65]. “Sense of collective belonging” is a localized item. Individualism is a fundamental feature of Western culture, and people with this cultural background are concentrated on “I”, while collectivist culture is a typical character in China, and individuals are more centered on “we” [58, 66]. When nurses provide psychological care services, their own sense of collective belonging has a profound impact on their job efficiency, which in turn affects the quality of nursing care. To fully explore parts below the iceberg, nursing managers should help nurses understand the characteristics and social value of psychological care, stimulate their motivation for personal growth and strengthen their sense of collective belonging.

## Implications for nursing, education, and future research

The results of this study provide a framework for curriculum development and performance assessment in PCC educational programs for nurses. This PCC framework applies to nonpsychiatric nurses because the psychological problems of patients in these departments are of concern. Therefore, nurse educators could use this framework to teach nurses after assessing the validity of cross-cultural adaptation in their respective nations. Furthermore, there is limited evidence as to the impact of nursing education programs on patient outcomes [67], and nursing work is needed to link competences to practice outcomes. Hence, nursing curricula should include educational outcomes that allow for an accurate evaluation of how these will relate to patient care. In the future, an assessment tool based on the PCC framework will be developed to assess the level of psychological care competency for nurses and the reliability and validity will be reported.

## Limitations and strengths

Our study has its own limitations. The gender of recruited participants disproportionately distributed because of the natural of nursing profession. In addition, the study results are highly contextualized. The participants were from a hospital or university setting, and the validated and adapted nursing competencies framework describes nursing practice in hospitals, which does not take into account community care practice. Further research is needed to confirm the transferability of the findings to community care practice.

Based on the Iceberg Model, the PCC framework for nurses is more focused and comprehensive, which can clarify their competency requirements to understand the weaknesses of nursing staff competency and provide targeted training. Moreover, a strength of our Delphi study was the involvement of a relevant national expert panel with significant experience in clinical psychological care. Their expertise allowed for in-depth reflection on the relevance of PCC in China. The study provides useful insights into nursing competences related to psychological care.

## Conclusion

This study used a literature review, semistructured interviews and the Delphi technique to develop a PCC framework for Chinese nurses. The results, which included 5 domains and 22 subdomains, were scientific and reliable. This framework could be used both to guide the design of the curricula and to actively involve nurses in self-assessment to understand needs for the development of psychological care. However, this PCC framework developed in this study has yet to be evaluated in clinical settings. An evaluation tool based on the PCC framework will be created in the future to analyze the reliability and validity of this framework. Furthermore, it is suggested that uniform training for nurses based on the PCC framework be conducted in healthcare institutions to improve PCC among nurses.

### Electronic supplementary material

Below is the link to the electronic supplementary material.

Supplementary Material 1

Supplementary Material 2

## Data Availability

Not applicable.
