# Supplementary figures and images for: Developing a psychological care competences framework for nurses in China: a mixed methods study
Source: BMC Nurs. 2024 Feb 19;23:129. doi: 10.1186/s12912-024-01778-3 (PMC10877790; doi:10.1186/s12912-024-01778-3)

**Appendix Ⅰ. Database searching**


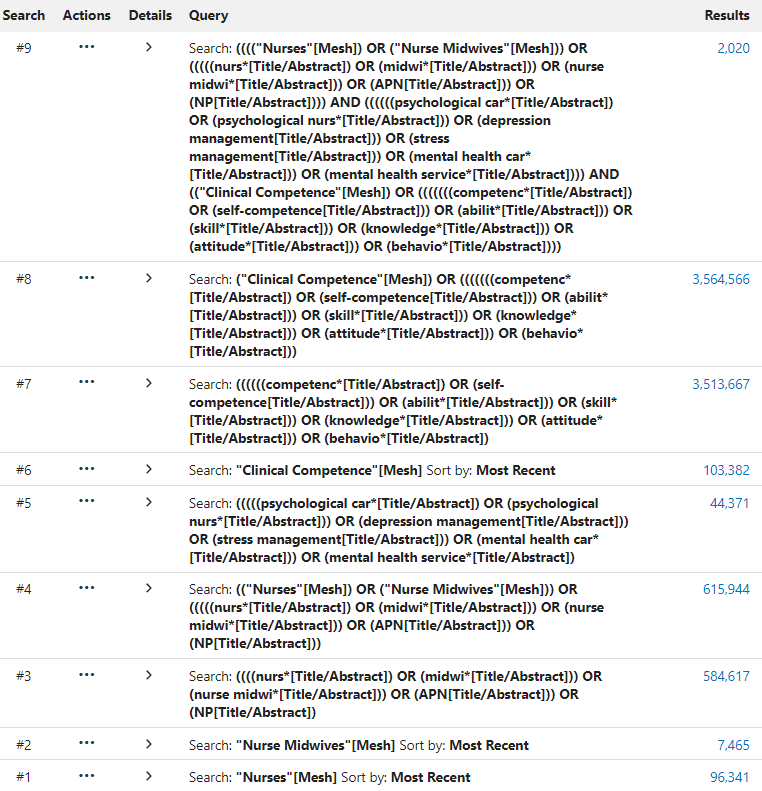

Supplement: Supplementary file 1 — Supplementary Material 1 [file 12912_2024_1778_MOESM1_ESM.docx]
