# Supplementary material for: Developing a psychological care competences framework for nurses in China: a mixed methods study
Source: BMC Nurs. 2024 Feb 19;23:129. doi: 10.1186/s12912-024-01778-3 (PMC10877790; doi:10.1186/s12912-024-01778-3)
Supplement: Supplementary file 2 — Supplementary Material 2 [file 12912_2024_1778_MOESM2_ESM.docx]

## Appendix II. The semi-structured interview guideline

**1. The semi-structured interview guideline for nursing managers included the following questions:**

(1) Could you please share your thoughts on psychological nursing?

(2) Could you please introduce the roles of nurses in the delivery of psychological nursing to patients?

(3) Could you please share one to two impressive events about psychological nursing implemented by clinical nurses? Maybe it was a success or a regret. How did these events arise? Who was involved? How did the nurse deal with it? What were the nurse’s thoughts and feelings at the time? How did things turn out?

(4) What knowledge, skills, and moralities do you think nurses should possess to be competent in psychological nursing? Could you please provide a specific explanation for each element?

(5) In addition to the above, what other competencies do you think nurses should possess to be competent in psychological nursing?

**2. The semi-structured interview guideline for clinical nurses included the following questions:**

(1) Could you please share your thoughts on psychological nursing?

(2) Could you please introduce your roles in psychological nursing to patients?

(3) Could you please share more than three impressive events you participated in during the psychological nursing? Maybe it was a success or a regret. How did these events arise? Who was involved? How did you deal with it? What were your thoughts and feelings at the time? How did things turn out?

(4) What knowledge, skills or moralities do you think need to be improved and developed in the process of psychological nursing? Could you please provide specific reasons?

(5) Do you have any other additions to the psychological nursing competence elements?
